# Sigma Receptor Ligands Prevent COVID Mortality In Vivo: Implications for Future Therapeutics

**DOI:** 10.3390/ijms242115718

**Published:** 2023-10-29

**Authors:** Reed L. Berkowitz, Andrew P. Bluhm, Glenn W. Knox, Christopher R. McCurdy, David A. Ostrov, Michael H. Norris

**Affiliations:** 1 Department of Pathology, Immunology and Laboratory Medicine, College of Medicine, University of Florida, Gainesville, FL 32610, USA; reedberkowitz@ufl.edu (R.L.B.); ostroda@pathology.ufl.edu (D.A.O.); 2 Spatial Epidemiology and Ecology Research Laboratory, Department of Geography, College of Liberal Arts and Sciences, University of Florida, Gainesville, FL 32611, USA; 3 Emerging Pathogens Institute, University of Florida, Gainesville, FL 32601, USA; 4 Department of Medicinal Chemistry, College of Pharmacy, University of Florida, Gainesville, FL 32610, USA; 5 Translational Drug Development Core, Clinical and Translational Sciences Institute, University of Florida, Gainesville, FL 32610, USA; 6 School of Life Sciences, University of Hawaiʻi at Mānoa, Honolulu, HI 96822, USA

**Keywords:** SARS-CoV-2, coronaviruses, COVID, antivirals, therapeutics, vaccines, lactoferrin, mutations, variants, sigma receptors, diphenhydramine, antiviral resistance, treatment strategies

## Abstract

The emergence of lethal coronaviruses follows a periodic pattern which suggests a recurring cycle of outbreaks. It remains uncertain as to when the next lethal coronavirus will emerge, though its eventual emergence appears to be inevitable. New mutations in evolving SARS-CoV-2 variants have provided resistance to current antiviral drugs, monoclonal antibodies, and vaccines, reducing their therapeutic efficacy. This underscores the urgent need to investigate alternative therapeutic approaches. Sigma receptors have been unexpectedly linked to the SARS-CoV-2 life cycle due to the direct antiviral effect of their ligands. Coronavirus-induced cell stress facilitates the formation of an ER-derived complex conducive to its replication. Sigma receptor ligands are believed to prevent the formation of this complex. Repurposing FDA-approved drugs for COVID-19 offers a timely and cost-efficient strategy to find treatments with established safety profiles. Notably, diphenhydramine, a sigma receptor ligand, is thought to counteract the virus by inhibiting the creation of ER-derived replication vesicles. Furthermore, lactoferrin, a well-characterized immunomodulatory protein, has shown antiviral efficacy against SARS-CoV-2 both in laboratory settings and in living organisms. In the present study, we aimed to explore the impact of sigma receptor ligands on SARS-CoV-2-induced mortality in ACE2-transgenic mice. We assessed the effects of an investigational antiviral drug combination comprising a sigma receptor ligand and an immunomodulatory protein. Mice treated with sigma-2 receptor ligands or diphenhydramine and lactoferrin exhibited improved survival rates and rapid rebound in mass following the SARS-CoV-2 challenge compared to mock-treated animals. Clinical translation of these findings may support the discovery of new treatment and research strategies for SARS-CoV-2.

## 1. Introduction

Sigma receptors are widely expressed intracellular chaperone proteins that typically reside on the endoplasmic reticulum (ER) membrane [1]. Sigma receptors exhibit a broad array of physiological properties with roles in mediating cell stress responses, nociception, and addiction [2,3,4]. Coronavirus-induced cell stress helps form an ER-derived complex conducive to virus replication [5]. Sigma receptors were unexpectedly linked to the SARS-CoV-2 life cycle because sigma receptor ligands exhibited direct antiviral activity [6].

Previously, we investigated the role of sigma-1 and sigma-2 receptors in mediating antiviral activity against SARS-CoV-2 in vitro [6]. These studies found that sigma-1 and sigma-2 receptor ligands exerted direct antiviral activity against SARS-CoV-2 in vitro. This observation was interpreted as the consequence of sigma receptor ligation on cell stress, inhibiting the formation of the coronavirus replication complex, thus, decreasing viral replication. Additionally, we previously investigated the effect of an investigational antiviral drug combination consisting of an off-target sigma receptor ligand antihistamine (diphenhydramine) and an immunomodulatory protein (lactoferrin) against SARS-CoV-2 in vitro. Diphenhydramine was selected due to the analysis of electronic health records revealing a reduced incidence of SARS-CoV-2 positivity in subjects taking antihistamines and its activity as an off-target sigma receptor ligand [7]. Lactoferrin was selected based on high-throughput drug repurposing screenings for SARS-CoV-2 antivirals as well as demonstrated in vitro activity against SARS-CoV-2 [8,9]. The diphenhydramine/lactoferrin combination was demonstrated to reduce SARS-CoV-2 replication in vitro by 99% [6].

**Figure 1:**
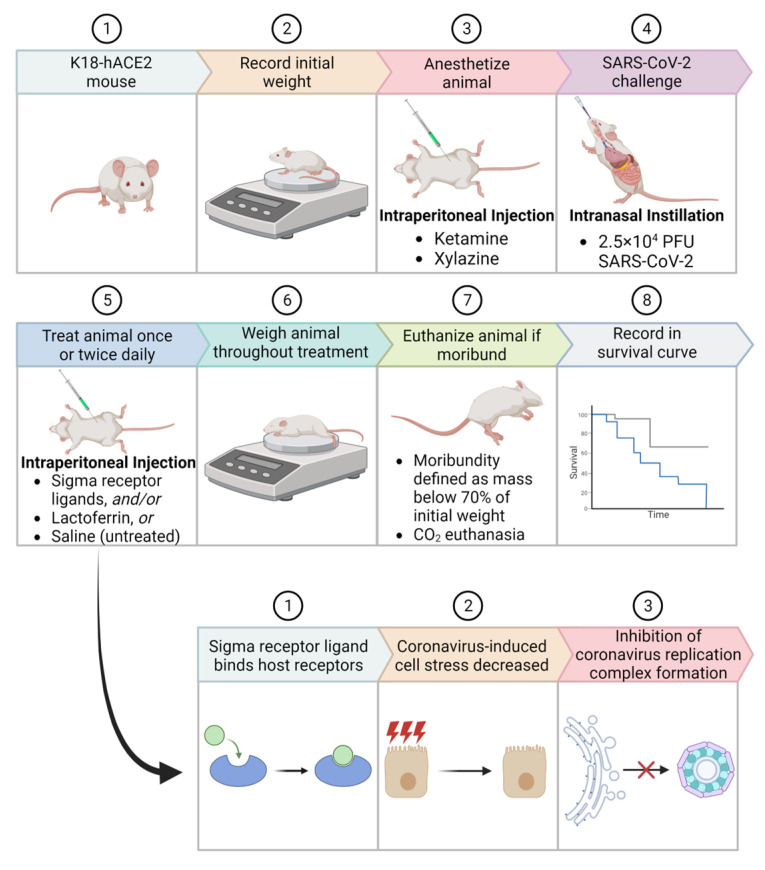
Schematic representation of survival studies in ACE2-transgenic mice post SARS-CoV-2 infection. (**Top**) The stepwise procedure entails: (1) Use of K18-hACE2 mouse model; (2) Recording of initial weight; (3) Anesthetizing with Ketamine and Xylazine; (4) Intranasal challenge with 2.5 × 10^4^ PFU SARS-CoV-2; (5) Daily treatments with Sigma receptor ligands, Lactoferrin, or Saline (untreated); (6) Continuous weight monitoring; (7) Euthanasia upon reaching moribund state, defined as a mass below 70% of initial weight; and (8) Documentation of survival curve. (**Bottom**) Proposed action mechanism of Sigma receptor ligands: (1) Binding to host receptors; (2) Reduction of coronavirus-induced cell stress; and (3) Inhibition of coronavirus replication complex formation.

In this study, we investigated the role of highly specific sigma receptor ligands on SARS-CoV-2-induced mortality in ACE2-transgenic mice. K18-hACE2 mice were selected for this study because they express human ACE2 receptors, effectively simulating the human response to SARS-CoV-2 and allowing evaluation of the therapeutic effect of sigma receptor ligands [10]. These mice are widely available, thoroughly studied as models of SARS-CoV-2 infection, and their broad expression of the hACE2 gene leads to acute infections [10,11]. The expression of human ACE2 also facilitates the assessment of spike-hACE2 interaction interference. In addition, we measured the effect of the diphenhydramine/lactoferrin combination on SARS-CoV-2-induced mortality in ACE2-transgenic mice. The effects of tested treatments on SARS-CoV-2 mortality in mice were determined by survival studies (Figure 1).

These data are relevant to the diminishing efficacy of available COVID drugs and potential strategies to improve efficacy, including the use of sigma receptor ligands. The potential of combinatorial therapies targeting multiple viral replication stages for enhanced efficacy against future coronaviruses and reduced susceptibility to declining efficacy compared to current COVID drugs is discussed.

## 2. Results

**Figure 2:**
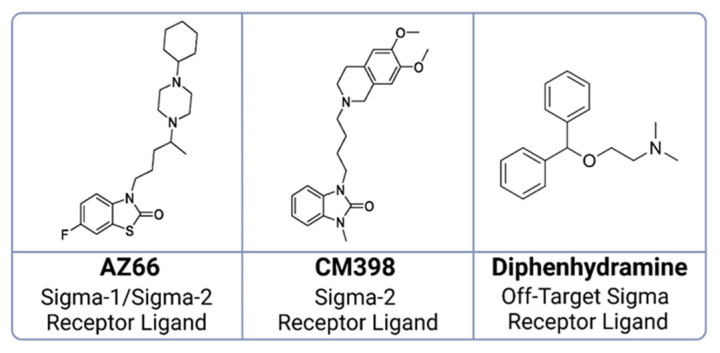
Chemical structures of ligands utilized in SARS-CoV-2 survival studies in mice. Displayed are the molecular structures of: AZ66, a dual sigma-1 and sigma-2 receptor ligand; CM398, specific to sigma-2 receptors; and diphenhydramine, recognized as an off-target sigma receptor ligand.

**Figure 3:**
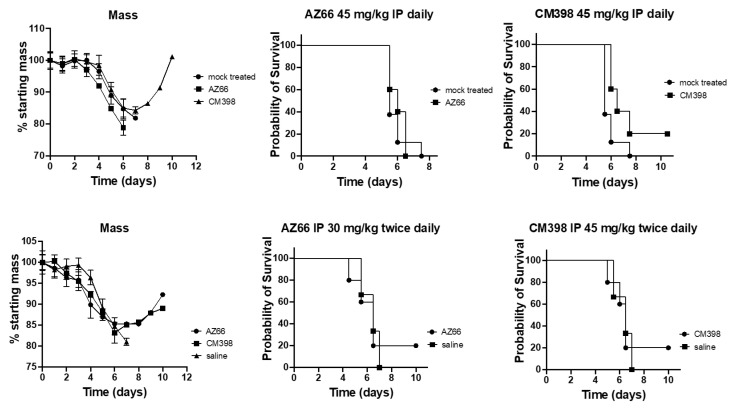
Efficacy of highly specific sigma receptor ligands on SARS-CoV-2 survival in ACE2-transgenic mice. The ACE2-transgenic mice underwent intranasal challenge with 2.5 × 10^4^ PFU SARS-CoV-2 (strain UF-1) and subsequently received treatment either with the vehicle control (mock-treated), CM398 (sigma-2 receptor-specific ligand), or AZ66 (dual sigma-1/sigma-2 receptor ligand). Two distinct dosing regimens were employed, intraperitoneal (IP) injection either daily or twice daily. The number of animals in the saline-treated group was 8, whereas the treated groups contained 5 animals each. The displayed results capture the survival outcome for each respective treatment.

K18-hACE2 transgenic mice were challenged with SARS-CoV-2 strain UF-1. Mice were treated daily or twice daily with either saline, a sigma-1 and sigma-2 receptor binding ligand (AZ66), a sigma-2 receptor binding ligand (CM398), or an investigational antiviral drug combination consisting of an off-target sigma receptor ligand (diphenhydramine; DPH) and an immunomodulatory protein (lactoferrin; LF) (Figure 2). All mice treated with only saline (mock-treated) or AZ66 underwent rapid weight loss, culminating in a 100% mortality rate, as illustrated in Figure 3. In contrast, mice treated with CM398 initially lost weight rapidly but subsequently displayed a sharp rebound in mass, exhibiting nonzero survival rates.

**Figure 4:**
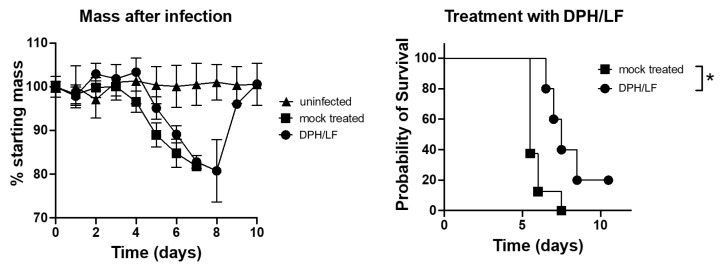
Impact of diphenhydramine/lactoferrin (DPH/LF) treatment on survival and weight loss in SARS-CoV-2-infected ACE2-transgenic mice. Mice were challenged with 2.5 × 10^4^ PFU SARS-CoV-2 (UF-1) intranasally and administered either vehicle control (mock treated) or a combination of 30 mg/kg diphenhydramine and 50 mg/kg lactoferrin (DPH/LF) twice daily via intraperitoneal injection. Group sizes: *n* = 3 (uninfected), *n* = 8 (mock-treated), and *n* = 5 (DPH/LF treated). Left panel: Percentage of starting mass post-infection over 10 days comparing uninfected, mock-treated, and DPH/LF-treated mice. Right panel: Probability of survival over 10 days for mock treated and DPH/LF-treated mice. An asterisk denotes a significant difference in outcomes between mock treated and DPH/LFN-treated groups (*p* ≤ 0.05).

In an additional survival study, K18-hACE2 transgenic mice were challenged with the SARS-CoV-2 strain UF-1. Mice were treated with saline (mock treated) or the investigational diphenhydramine/lactoferrin combination. The survival study presented in Figure 4 unequivocally demonstrates that employing the diphenhydramine/lactoferrin combination significantly extends the survival time of COVID-infected mice with several achieving recovery (Figure 4). Notably, mice treated with a combination of diphenhydramine and lactoferrin exhibited the most gradual weight loss among all groups, followed by a pronounced rebound in weight and improved (nonzero) survival rates. This underscores the efficacy of the treatment in not only enhancing survival but also in moderating the rate of weight loss and facilitating rapid mass recovery for those who survived, outperforming all other treated and untreated groups in this regard.

**Figure 5:**
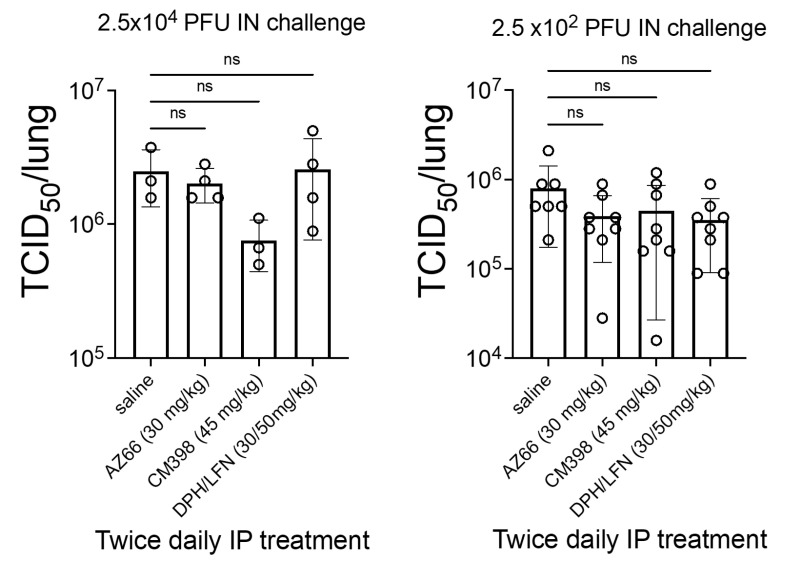
Quantitation of SARS-CoV-2 virus particles in the lungs of treated ACE2-transgenic mice. Mice were intranasally challenged with 2.5 × 10^4^ PFU or 2.5 × 10^2^ PFU SARS-CoV-2 (UF-1) and then treated with the vehicle control (saline), CM398 (sigma-2 receptor-specific ligand), AZ66 (sigma-1/sigma-2 receptor ligand), or diphenhydramine (sigma receptor ligand) combined with lactoferrin. The 50% tissue culture infectious dose (TCID_50_) was calculated based on lungs harvested 48 h after infection. An “ns” indicates non-statistically significant differences between treatment and placebo groups. Circles represent individual animals.

No statistically significant difference was detected in a 50% tissue culture infectious dose (TCID_50_) in treated mice 48 h after viral challenge compared to mock-treated mice (Figure 5). This suggests that immunomodulatory mechanisms or antiviral effects in other sites of the animal contributed to improved survival in treated mice rather than a direct antiviral effect in the lungs.

## 3. Discussion

### 3.1. Future of Coronavirus and COVID Drugs

**Figure 6:**
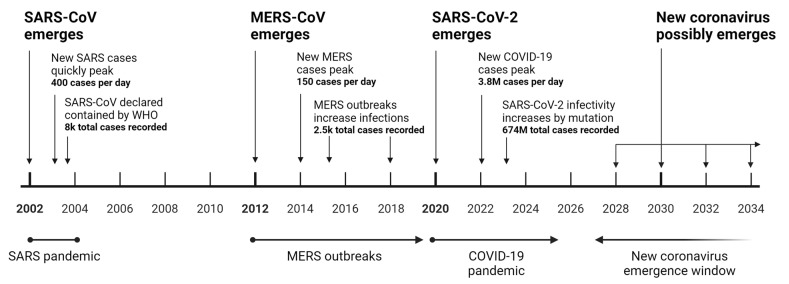
Historical and potential emergence of lethal coronaviruses. This timeline illustrates the sequential emergence of significant coronaviruses from 2002 to a projected window up to 2034. In 2002, SARS-CoV emerged, quickly reaching a peak of 400 cases per day, and was declared contained by the WHO after 8000 total cases. MERS-CoV peaked at 150 new cases per day, with subsequent outbreaks leading to 2500 total infections. SARS-CoV-2, causing the COVID-19 pandemic, had a daily peak of 3.8 million cases, accumulating 674 million cases globally. The period from 2028 to 2034 represents the anticipated window for the potential emergence of a new lethal coronavirus. The timeline underscores the cyclical nature of coronavirus outbreaks, highlighting the importance of global preparedness for future occurrences.

Coronaviruses of concern have emerged periodically since 2002 [12,13,14]. Based on this pattern, considering the continued lack of effective surveillance and prevention measures in Asian wet markets [15], it is prudent to anticipate new lethal coronavirus outbreaks with the potential to emerge in the near future (Figure 6). Although the infectious mechanism of the next coronavirus has yet to be revealed, there is a significant likelihood that it may have similar infectious advantages that were seen in SARS-CoV-2, such as the furin cleavage site.

It is important to plan ahead for drug development directed at the next emerging coronavirus, but it is challenging given that the virus sequences and host cell receptors are not known. Thus, in preparation, healthcare professionals may benefit by focusing on mechanisms of action conserved in all coronaviruses rather than by targeting proteins unique to SARS-CoV-2.

### 3.2. Current Tools against COVID, and Their Pitfalls

Small molecule antivirals, monoclonal antibodies, and vaccines are available but have demonstrated limitations in terms of effectiveness and safety. As SARS-CoV-2 continues to mutate, the efficacy of current therapeutics is expected to drop. For example, vaccine efficacy in terms of CFR reduction in the United States dropped from 90.5% pre-Delta to 72.7% following the emergence of Omicron [16]. Despite the decreasing overall COVID-19 CFR, the high levels of vaccine specificity resulted in a quickly diminishing efficacy. Vaccine manufacturers also struggle with long regulatory approval and manufacturing timelines that prevent updated vaccines from reaching the population in a timely manner. For example, the first SARS-CoV-2 vaccine updated for the Omicron variant was approved for use in the United States by September 2022 [17], about 10 months after Omicron emerged [18]. Additionally, drugs that bind in a highly specific manner to distinct viral protein structures have resulted in the selection of virions with mutations that result in evasion of antiviral effect. 

Paxlovid (nirmatrelvir/ritonavir), a viral protease inhibitor drug combination, has flaws that could jeopardize its widespread use. Paxlovid patients commonly experience “COVID rebound”, a phenomenon in which SARS-CoV-2 replication will initially decrease, but then return to normal, causing a rebound in symptomatic disease [19]. 

One theory behind the mechanism of Paxlovid rebound is that virions are pressured to develop Paxlovid-resistant protease mutations, thus, creating Paxlovid-resistant strains of SARS-CoV-2. While the theory has not been confirmed in clinical settings, two studies (one preprint and one peer-reviewed) showed that SARS-CoV-2 treated with Paxlovid in vitro resulted in mutations at the same protease positions [20,21]. These mutations resulted in an 80-fold decrease in Paxlovid efficacy in vitro. Spontaneously occurring mutations of the protease in circulating variants have also decreased Paxlovid efficacy [22], demonstrating that regardless of the mechanism for gaining resistance (i.e., as a result of Paxlovid use or spontaneous mutations), decreased efficacy would still occur if the antiviral drugs induced the selection of resistant variants. Accordingly, protease inhibitors developed for viruses like HCV and HIV have had their own troubles maintaining efficacy due to resistance [23]. 

SARS-CoV-2-specific nucleotide analogs that function by inducing random viral mutations can also result in onward transmission of mutated viruses. A 2023 article found a specific class of phylogenetic branches that emerged in 2022 following the introduction of molnupiravir [24]. Elevated G-to-A and C-to-T mutation rates were observed in these branches, corresponding to the mechanism of action of molnupiravir [25]. These data indicates that molnupiravir use, like Paxlovid, could result in new resistant SARS-CoV-2 variants exhibiting drug-induced mutations. While mutations caused by nucleotide analogs usually harm viruses or leave them unaffected [26], it is possible that these mutations could affect the efficacy of antivirals that bind to distinct viral protein structures (if such structures are mutated by molnupiravir use). 

The most exquisitely specific COVID therapies developed to date are likely to be monoclonal antibodies. When newly developed, monoclonal antibodies exhibit dramatic antiviral effects and acceptable safety profiles. However, the use of monoclonal antibodies to treat COVID has halted because mutations in new variants resulted in a profoundly decreased efficacy [27,28]. It seems unlikely that an existing monoclonal antibody will be effective against a distinct new coronavirus outbreak.

Due to the ever-decreasing drug efficacy from drug-induced and spontaneous mutations, continuing to develop variant-specific SARS-CoV-2 antiviral drugs may not be the optimal approach. The strategies most likely to succeed against future SARS-CoV-2 variants, and other new coronaviruses yet to emerge, should inhibit the virus life cycle at multiple steps common to known coronaviruses.

### 3.3. Repurposed Drugs That Inhibit the Coronavirus Life Cycle

Repurposing of FDA-approved drugs is a promising strategy for the rapid identification of treatments due to known safety profiles, robust supply chains, and short deployment time frames. In an effort to identify drugs that can inhibit a SARS-CoV-2 infection, a landmark study generated a protein interaction map as the basis for target identification and drug repurposing [29]. The study found 69 small molecule compounds that could inhibit SARS-CoV-2 infection in vitro, 29 of which were FDA-approved drugs. 

**Figure 7:**
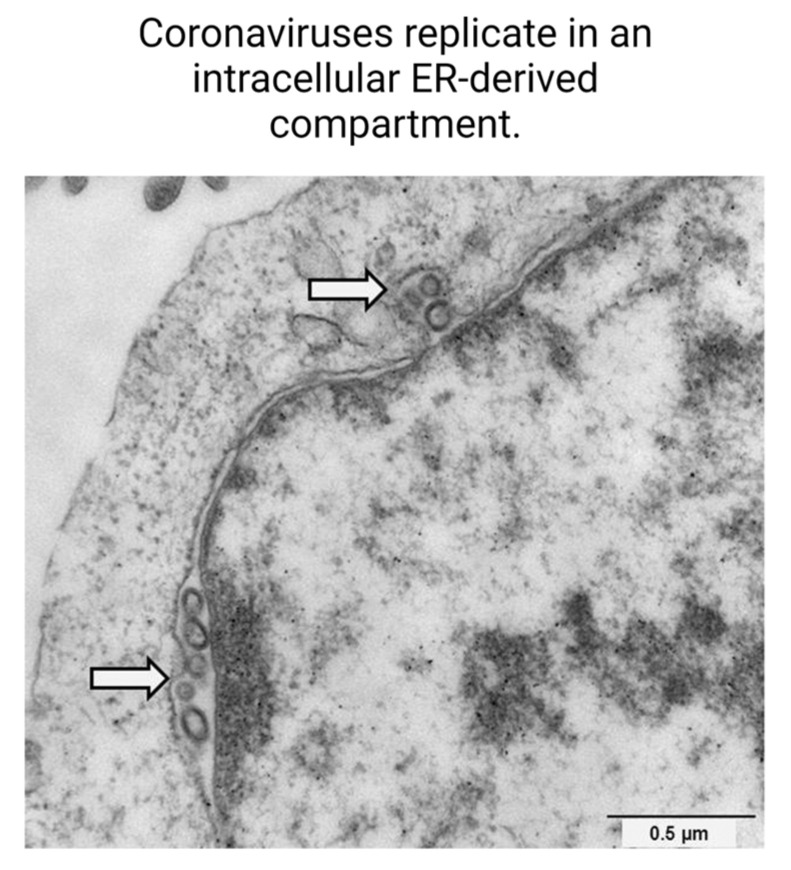
Coronaviruses replicate in an intracellular ER-derived compartment. Electron micrograph depicting the intracellular replication sites of coronaviruses within ER-derived compartments, as highlighted by arrows. Scale bar represents 0.5 µm [32].

The study found that many pharmacological agents that displayed antiviral activity were known or predicted ligands of the sigma-1 and sigma-2 receptors, which are ER membrane proteins that participate in the modulation of cell stress. Coronaviruses, including SARS-CoV-2, replicate in a membranous compartment derived from the ER (Figure 7) [30]. These compartments provide developing virions with an environment favorable to RNA synthesis and protection from host cell immune functions [31]. Coronaviruses are known to cause host cell ER stress and activate pathways to facilitate adaptation of the host cell machinery to viral needs [5]. Virus-induced stress helps form the compartment within cells conducive to SARS-CoV-2 replication. Drugs that bind sigma receptors are thought to exert antiviral activity by inhibiting the formation of this intracellular compartment where coronavirus replication occurs [6].

It was demonstrated that specific FDA-approved antihistamines exhibit off-target sigma receptor binding activity and antiviral activity against SARS-CoV-2 [6]. For example, clemastine, cloperastine, astemizole [29], hydroxyzine, azelastine, and diphenhydramine [7] are common antihistamines with direct antiviral activity in vitro. Consistent with these data, electronic health records showed that usage of antihistamines, including diphenhydramine, was associated with a decreased incidence of SARS-CoV-2 positivity [7].

In this study, we showed that highly specific sigma receptor ligands protected ACE2-transgenic mice from SARS-CoV-2-induced mortality.

Finding drug combinations with broad activity against coronaviruses is an important approach to strengthen the toolkit for SARS-CoV-2 and future coronaviruses. For SARS-CoV-2, all variants replicate using the same ER-derived compartment. The same is true of all coronaviruses [30], therefore, increasing the likelihood that sigma-receptor-targeting therapeutics will be effective against future variants and novel coronaviruses.

### 3.4. Antiviral Drug Combinations for COVID

Antiviral combinations targeting highly conserved viral mechanisms have proven their success in treating drug-resistant viruses. For example, HIV and hepatitis C patients are treated with drug combinations instead of single drugs, with each drug targeting distinct pathways involved in the virus life cycle [33,34]. For SARS-CoV-2, effective antiviral drug combinations may consist of a sigma receptor-binding drug combined with an agent that interferes with other aspects of the virus life cycle. The most rapidly employable antiviral drug combination would include drugs that are economical, stable, and have a long history of safety.

**Figure 8:**
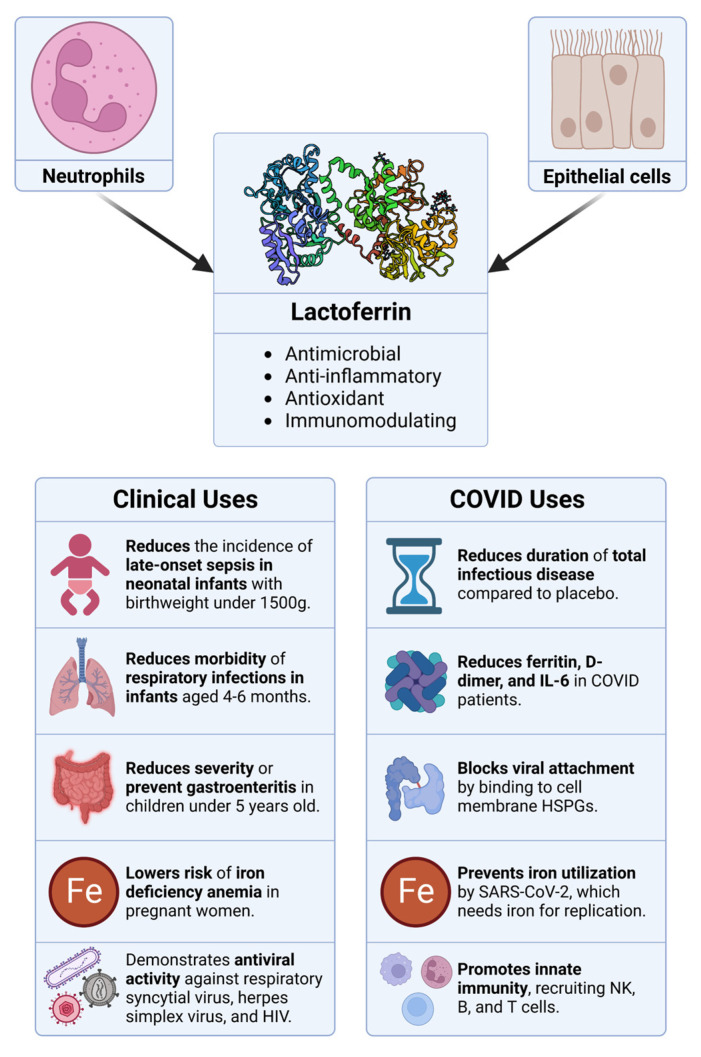
Overview of the sources, properties, and therapeutic applications of lactoferrin. Originating from neutrophils and epithelial cells, lactoferrin boasts a range of bioactivities, including antimicrobial, anti-inflammatory, antioxidant, and immunomodulatory properties. The clinical utility of lactoferrin spans from managing neonatal infections to mitigating specific symptoms in COVID-19 patients, among other therapeutic applications.

One such drug is diphenhydramine, an over-the-counter antihistamine that is a potentially useful off-target sigma receptor ligand in an antiviral drug combination due to its wide accessibility and long safety record [35]. Diphenhydramine has also been previously researched for repurposing as an antiviral drug for filoviruses including the Ebola virus and Marburg virus [36]. A drug that would complement sigma receptor ligands by targeting a different part of the virus life cycle could result in a combination that is highly efficacious against COVID. Another therapeutic option is lactoferrin, which is a large iron-binding glycoprotein that is thought to have important properties in terms of antimicrobial activity and modulation of the immune system (Figure 8) [37]. It is produced by secretory cells in all mammals and is expressed at the highest level in breast milk. Lactoferrin is classified as a “Generally Recognized As Safe” dietary supplement by the FDA and has been shown to be safe in clinical studies in the context of neonates, children, and adults (Figure 8) [38]. Lactoferrin has been shown to display broad antiviral activity, including against rotavirus, respiratory syncytial virus, herpes virus, and HIV [39,40]. A high-throughput screen of 1425 compounds that sought to discover FDA-approved drugs with activity against SARS-CoV-2 in vitro led to the discovery of lactoferrin as the most efficacious hit [8]. 

Both diphenhydramine and lactoferrin are safe for oral consumption by adult humans. Effective doses of diphenhydramine for its indicated use (allergy symptoms) are 25 to 50 mg every 4 to 6 h as needed, not to exceed 300 mg/day [35]. Lactoferrin does not have established dosage guidelines but has demonstrated safety in clinical trials with doses of up to 7200 mg/day for 8 weeks and 3000 mg/day for 12 months [41,42].

Lactoferrin has been tested in the clinic for the treatment of COVID because of its antiviral activity, immunomodulatory properties, and safety profile. Such studies have garnered highly varied and controversial results, ranging from no observed effect to symptom resolution in all treated patients [43,44,45,46,47]. This variation can likely be attributed to methodological differences, including but not limited to the delivery method (intranasal spray or oral), type (liposomal or non-liposomal lactoferrin), endpoints, dosage, time of dosage relative to illness onset, and in one study, use of anti-inflammatory adjuvants. With only a 30% reduction in viral replication in vitro, it is not expected that treatment solely with lactoferrin would translate to clinical success in SARS-CoV-2 patients. However, combining lactoferrin with diphenhydramine has been shown in vitro to significantly improve its antiviral efficacy against SARS-CoV-2 [6].

**Figure 9:**
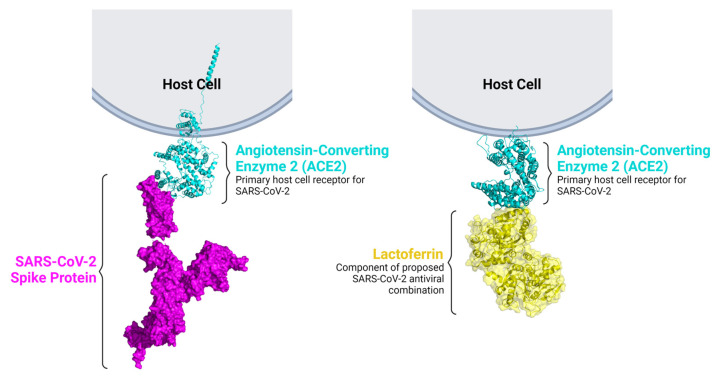
Lactoferrin binds ACE2, the host receptor for SARS-CoV-2. A model is shown in which lactoferrin (yellow) interacts with ACE2 (cyan) receptor, hindering the binding of the SARS-CoV-2 spike protein (magenta) to ACE2. This mechanism suggests a reduction in the ability of the virus to infiltrate the host cell.

An investigational antiviral drug combination effective against SARS-CoV-2 in vitro has been described in which each component is an approved agent inhibiting distinct mechanisms of the virus life cycle. Diphenhydramine, an off-target sigma receptor ligand, is thought to exert antiviral activity by inhibiting the host cell stress response and thereby inhibiting the formation of the ER-derived coronavirus replication compartment. Diphenhydramine was combined with lactoferrin which is thought to exert indirect antiviral activity via immunomodulation as well as direct antiviral activity by binding heparan sulfate proteoglycans (HSPGs) [45] and angiotensin-converting enzyme 2 (ACE2) [48], the host cell receptors of SARS-CoV-2 (Figure 9) [45].

**Figure 10:**
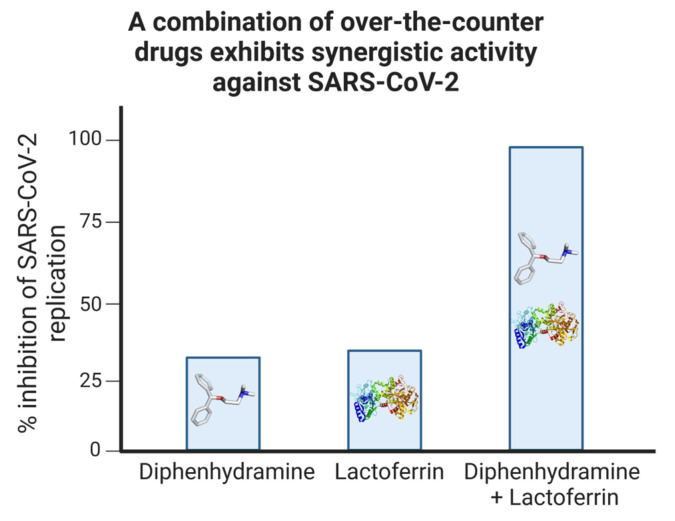
Synergistic inhibition of SARS-CoV-2 replication by a combination of non-prescription drugs. Vero E6 cells were subjected to infection with a SARS-CoV-2 isolate and subsequently treated with either diphenhydramine, lactoferrin, or a combination of both. The replication efficiency was evaluated by quantifying the SARS-CoV-2 genomic presence using PCR. Adapted with permission from Ref. [6]. 2021, MDPI.

Diphenhydramine and lactoferrin were tested in combination to determine any enhanced antiviral activity. Independently, diphenhydramine and lactoferrin reduced SARS-CoV-2 replication by approximately 30% in vitro. Strikingly, in combination, diphenhydramine and lactoferrin exhibited a synergistic effect in vitro, resulting in a 99% reduction in SARS-CoV-2 replication in monkey and human cell lines (Figure 10) [6]. In vivo, we showed that ACE2-transgenic mice challenged with SARS-CoV-2 demonstrated significantly improved survival with sigma receptor ligand treatment.

It follows that the in vivo results in this study of SARS-CoV-2-challenged ACE2-transgenic mice treated with the diphenhydramine/lactoferrin combination exhibited the slowest decline in mass compared to any other group, nonzero survival rates compared to untreated mice, and a rapid rebound in mass in surviving mice. However, a direct antiviral effect was not observed in vivo. This may be explained by the effects of the combination on immunomodulation. 

COVID is a multiphasic disease in which initial symptomatic manifestations are caused by the “viral phase”, and later symptomatic manifestations, including mortality, are caused by the “inflammatory phase”, which includes lethal cytokine storms [49]. Thus, it is posited that the protective effects of the diphenhydramine/lactoferrin combination observed in this study are mediated by the prevention of lethal inflammation. 

Additionally, therapeutic effect may have been achieved by direct antiviral effect in non-lung sites of the animal, or antiviral effect after the viral phase of the disease which may not have been observed at the time of the predetermined endpoint study.

## 4. Materials and Methods

### 4.1. Virus Culturing and Growth

SARS-CoV-2 work was performed in a biosafety level 3 (BSL-3) lab using practices and procedures approved by the University of Florida Institutional Biosafety Committee. SARS-CoV-2 strain UF-1 was obtained from a patient at University of Florida Shands Hospital (Gainesville, FL, USA) as previously described [6,50]. SARS-CoV-2 was cultured in Vero E6 cells grown in DMEM + 2% heat-inactivated fetal bovine serum (FBS) with PenStrep at 37 °C in a 5% CO_2_ environment also as previously described. Vero E6 cells were obtained from ATCC (Manassas, VA, USA). Viral stocks were harvested and quantified using TCID_50_ dilution titering in Vero E6 cells.

### 4.2. Sigma Ligands and Other Chemicals Used in This Study

Sigma ligands were produced in the McCurdy lab at the University of Florida (Gainesville, FL, USA) as previously described [51,52,53,54]. Lactoferrin at 95% purity from cow milk was kindly donated by Milk Specialties Global (Eden Prairie, MN, USA). Diphenhydramine HCl at 98% purity was obtained from Spectrum Pharmaceuticals (Boston, MA, USA).

### 4.3. Animal Challenge Experiments

Animal work was carried out in the Animal Biosafety Level 3 (ABSL3) laboratory and was approved by the University of Florida Institutional Animal Care and Use Committee (IACUC) under IACUC protocol number 202111322. Six- to eight-week-old female K18-hACE2 mice were purchased from The Jackson Laboratory (Bar Harbor, ME, USA). K18-hACE2 mice are transgenic for the human ACE2 receptor and express it from the keratin 18 promoter to direct ACE2 expression to epithelia including airway epithelia. The mouse strain has been extensively characterized as a SARS-CoV-2 animal model, as their tissue-wide expression of human ACE2 ensures fulminant acute infections [10,11,55]. Mice were provided food and water ad libitum and housed in isolator caging. On the day of the challenge, the mice were weighed and then anesthetized by intraperitoneal (IP) injection of 100 μL of ketamine/xylazine (87.5/12.5 mg/kg) in saline. Fully anesthetized mice were challenged by intranasal instillation with 25 μL containing 2.5 × 10^4^ plaque-forming units (PFU) of SARS-CoV-2. Each day thereafter, mice were observed for moribundity, and the mass of each mouse was recorded. Treatments were delivered by an IP injection of the drugs suspended in 100 μL of saline, or saline alone. Treatments were given once or twice daily depending on the experiment, and the doses were titrated based on previously reported drug pharmacokinetic profiles [51,53]. Mice were determined to be moribund if their mass fell below 70% of their starting mass and were euthanized with carbon dioxide. 

### 4.4. Modeling Interactions between SARS-CoV-2 Spike Protein and Ligands

The model of the SARS-CoV-2 spike protein binding to ACE2 is based on the crystal structure of the SARS-CoV-2 spike protein complexed with ACE2 [56] (PDB 6M17). A structural model of ACE2 bound to lactoferrin was generated by HDOCK [57] using crystal structures of ACE2 [58] (PDB 1R42) and bovine lactoferrin [59] (PDB 1BLF).

## 5. Conclusions

**Figure 11:**
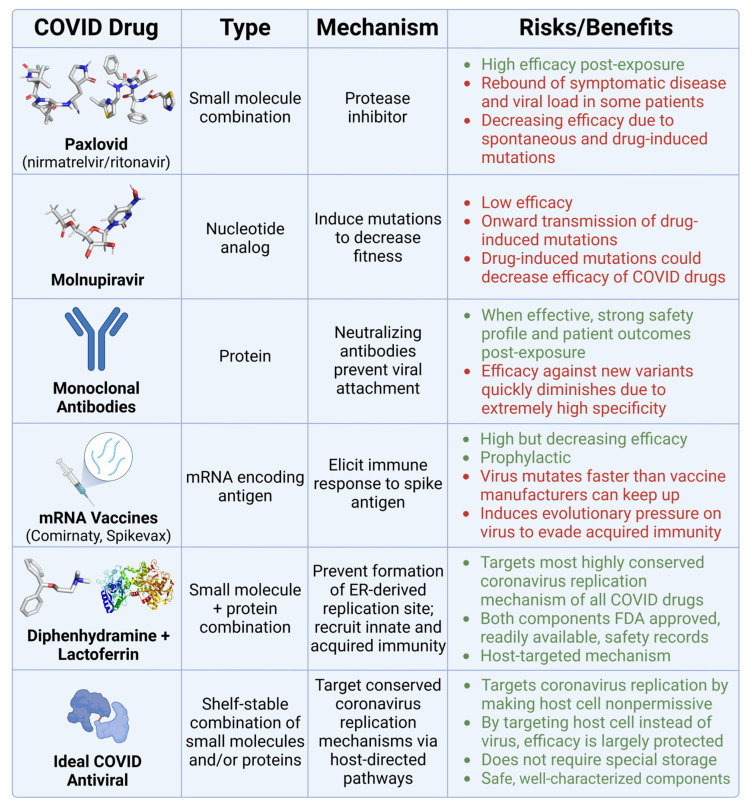
Comparison of COVID therapeutics. This table presents various COVID-19 drugs and their corresponding classification, mechanism of action, and associated risks (highlighted in red) and benefits (highlighted in green).

The current toolkit of COVID-specific antivirals is losing efficacy over time. Thus, further development of highly variant-specific antiviral drugs could be questioned. One option is to phase out currently available post-exposure COVID antiviral drugs due to the risks outlined in Figure 11.

There are benefits to updating the COVID antiviral toolkit with broader-spectrum antiviral combination drugs targeting multiple distinct mechanisms in the life cycle that are shared by all coronaviruses. This strategy will likely increase drug efficacy while decreasing the risk that drug-induced mutations, natural mutations, or new coronaviruses will render such an antiviral combination ineffective. Ideally, new antiviral drug combinations will function by making host cells nonpermissive to replication, a strategy that has proven to be effective in preventing antiviral resistance [60]. 

Sigma receptor ligands offer a promising approach to host-targeted antiviral activity against coronaviruses by inhibiting the formation of the coronavirus replication complex from the host endoplasmic reticulum. SARS-CoV-2-challenged ACE2-transgenic mice demonstrated improved survival when treated with highly specific sigma receptor ligands. Mice that survived were shown to rapidly rebound in mass following treatment. The sigma-2 receptor ligand CM398 showed a stronger effect than the sigma-1 receptor ligand AZ66.

**Figure 12:**
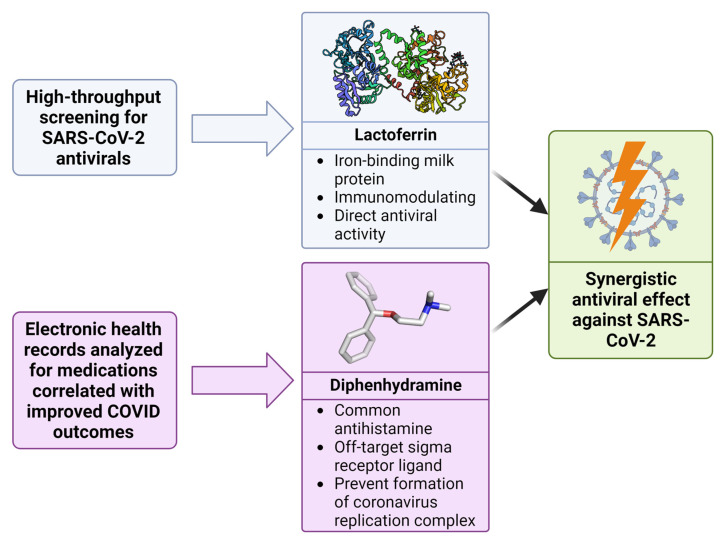
Discovery of synergy between lactoferrin and diphenhydramine against SARS-CoV-2. Utilizing high-throughput screening and analysis of electronic health records, both lactoferrin and diphenhydramine were identified as potential antiviral agents. When combined, these drugs demonstrate a potentiated, synergistic effect against SARS-CoV-2.

A current candidate fitting these criteria most likely to be effective for a new coronavirus antiviral combination may be the diphenhydramine/lactoferrin combination (Figure 12). Mice treated with this combination exhibited the slowest decline in mass following the challenge with SARS-CoV-2, improved survival rates, and rapid rebound in mass in surviving mice. Despite the lack of a statistically significant direct antiviral effect in the lungs of mice, improved outcomes were demonstrated, likely due to the previously outlined immunomodulatory effects of the combination. 

Both components of the combination exhibit direct antiviral activity in vitro as well as protective effects in vivo, are FDA-approved, economical, and have long histories of safety. Clinical investigation of the diphenhydramine/lactoferrin combination is warranted to determine benefits for COVID patients. The collection of clinical data pertaining to the efficacy or absence thereof resulting from the employment of the diphenhydramine/lactoferrin combination would be significantly advantageous if communicated by physicians. Such data could help to further inform coronavirus drug development strategies while potentially enabling the widespread employment of the diphenhydramine/lactoferrin combination if proven effective in clinical settings.

## Data Availability

All data generated and analyzed in the study are present within the figures and text of the article.
